# Factors associated with objective and subjective cognitive impairment in Chinese patients with acute major depressive disorder

**DOI:** 10.1186/s12888-023-04857-y

**Published:** 2023-05-19

**Authors:** Na Zhu, Jie Tong, Yu Pei, Jie Zhang, Xirong Sun

**Affiliations:** Shanghai Pudong New Area Mental Health Center, School of Medicine, Tongji University, Shanghai, China

**Keywords:** Depressive disorder, THINC-it, Cognitive impairment, Influencing factors

## Abstract

**Background:**

Patients diagnosed withmajor depressive disorder (MDD) usually experience impaired cognitive functioning, which might negatively impact their clinical and functional outcomes. This study aimed to investigate the association of specific clinical factors with cognitive dysfunction in a group of MDD patients.

**Methods:**

A total of 75 subjects diagnosed with recurrent MDD were evaluated during the acute stage. Their cognitive functions were assessed using the THINC-integrated tool (THINC-it) for attention/alertness, processing speed, executive function, and working memory. Clinical psychiatric evaluations, such as the Hamilton Anxiety Scale (HAM-A), the Young Mania Rating Scale (YMRS), the Hamilton Depression Scale (HAM-D), and the Pittsburgh Sleep Quality Index(PSQI), were used to assess patients’ levels of anxiety, depression and sleeping problems. The investigated clinical variables were age, years of education, age at onset, number of depressive episodes, disease duration, presence of depressive and anxiety symptoms, sleep problems, and number of hospitalizations.

**Results:**

The results revealed that significant differences were observed between the two groups in the THINC-it total scores, Spotter, Codebreaker, Trails, and PDQ-5-D scores (*P* < 0.001). The THINC-it total scores, Spotter, Codebreaker, Trails, and Symbol Check were significantly associated with age and age at onset(*P* < 0.01). In addition, regression analysis found that years of education was positively associated with the Codebreaker total scores (*P* < 0.05). the THINC-it total scores, Symbol Check, Trails, and Codebreaker were correlated with the HAM-D total scores(*P* < 0.05). Additionally, the THINC-it total scores, Symbol Check, PDQ-5-D and Codebreaker significantly correlated with the PSQI total scores (*P* < 0.05).

**Conclusion:**

We found a significant statistical association between almost all cognitive domains and different clinical aspects in depressive disorder, such asage, age at onset, severity of depression, years of education, and sleep problems. Additionally, education was shown to be a protective factor against processing speed impairments. Special considerations of these factors might help outline better management strategies to improve cognitive functions in MDD patients.

## Introduction

The World Health Organization estimates that depression is a leading cause of disability, as it affects more than 300 million people worldwide [1]. The clinical manifestations of major depressive disorder (MDD) are not limited to mood symptoms but also include a wide range of cognitive and physical symptoms. Approximately two-thirds of people with depression have deficits in cognitive abilities [2, 3]. Several recent studies have suggested that cognitive dysfunction may persist despite symptomatic remission [4, 5], contributing to occupational and social difficulties [6–8]. Therefore, the study of cognitive impairment is of great significance for treating patients with MDD and improving their outcomes.

Suciu et al. [9] reported that patients who presented with a greater number of depressive episodes displayed executive dysfunctions, severe depression, and psychotic symptoms, which were associated with a negative influence on psychomotor speed evaluation. Lai et al. [10] found that patients with MDD manifested significant clinical MATRICS Consensus Cognitive Battery(MCCB) cognitive impairments. Given their differences in study design, such as the type of assessment tools used, the results andconclusions of these studies were not entirely consistent. Presently, the commonly used assessment instruments are limited by high cost, time consumption and complex use. Furthermore, selecting the optimal tool from the tremendous number of available tools remains challenging, thus limiting their clinical applicability. Comparatively, the THINC-it tool had the advantages of being quick, simple, and free, and was used to evaluate cognitive impairment with the use of short exercises and instructions in the present study, thereby potentially reducing unnecessary medical expenditures. It was the first tool to provide both subjective and objective assessments of cognitive impairment [11]. Thus, the THINC-it has been validated and found to be reliable in patients with depression at home and abroad [12–14], making it feasible for large-scale implementation.

In addition, patients’ quality of life is seriously affected by cognitive decline, which makes it difficult for them to return to society. Cognitive function consists of subjective and objective cognitive impairment, objective cognitive impairment refers to the pathological process associated with the abnormal processing of advanced brain intelligence related to learning,

memory, and thinking judgment. Also, subjective cognitive impairment [15] is defined by cognitive deficits subjectively perceived by individuals who have normal performance in objective neuropsychological examinations. cognitive impairment could be differentially affected by different clinical variables, such as years of education, job status, severity of depressive episodes, age at onset, job status, and physical activity performance [16, 17], However, the above studies did not break down the influencing factors of subjective and objective cognitive impairment. Recently, some scholars have refocused on this field and implemented the THINC-it tool to investigate some clinical factors (e.g.self-reported anxiety, psychosocial Function, perceived sleep quality) related to subjective and objective cognitive impairment [18–20]. However, much of these data were based on Western populations, and cannot be generalized to Chinese populations due to differences in treatments, prevalence patterns, and cultural perceptions of MDD. Given the influence of cultural, Western populations will express their concerns and seek help to actively change the current situation. Our social culture still stigmatizes depression and other mental health issues, unwilling to accept and face them, which brings some difficulties to implementation progress. To date, the validity and reliability of the THINC-it tool had been studied in depressed populations, but it had yet to be applied extensively to Chinese populations. Thus, it was used herein to determine the clinical factors that influence cognitive impairment. Taking a proactive approach to cognitive impairment, as well as related influencing factors, is conducive to early intervention and treatment, further enhancing long-term health benefits. These findings will also be helpful for future outlining better management strategies to improve cognitive functions in Chinese patients with MDD.

## Methods

The study group comprised 75 adults recruited from the Shanghai Pudong Area Mental Health Center (Shanghai, China) who were experiencing an acute depressive episode. All subjects, irrespective of gender, were aged between 18 and 60 years old and had at least eight years of education. The patients were diagnosed with recurrent MDD and major depressive episodes based on the ICD-10. An additional inclusion criterion was a Hamilton Depression Rating Scale (HAM-D_17_) score > 7 and a Young Mania Rating Scale (YMRS) score < 6. Patients who met the criteria for mental retardation, dementia, chronic alcoholism or any other substance dependence, history of head trauma, or current medical condition that could interfere with the level of cognitive performance, i.e.,consumption of alcohol within 8 h and use of benzodiazepines within 12 h of the THINC-it tool administration, and had been treated with electroconvulsive therapy (ECT) in the previous 6 months were excluded.

A total of 100 healthy controls were also recruited in the Pudong New Area of Shanghai, including college students, employees, teachers, freelancers, and social workers. The healthy control (HC) group were matched with the MDD in gender, age, education, and demographic data. The healthy controls had no diagnosis of any mental disorder according to the ICD-10 diagnostic criteria and did not have any history of neurological disorders, alcohol dependency, or family history of mental disorders among their first-degree relatives.The trial protocol was registered with ClinicalTrials.gov with the number NCT05053204.

### Clinical assessment

Before the start of the study, assessors were trained on the consistency of the THINC-it and scale evaluation, and the tests were performed after they passed the training. The evaluation was continuously performed in a quiet environment.

A cross-sectional research design was employed in this study. Data on the 75 MDD patients’ demographics and clinical characteristics, including age, sex, education level, marital status, age at onset, number of depressive episodes, presence of depressive and anxiety symptoms, disease duration, and number of hospitalizations, as well as the demographic and clinical characteristics of the 100 healthy volunteers were recorded and assessed. MDD and HC subjects were requested to complete a full set of cognitive assessments, namely, the THINC-it, and clinical psychiatric evaluations, such as HAM-A, YMRS, HAM-D, and Pittsburgh Sleep Quality Index(PSQI). By using the YMRS, patients who have not been diagnosed clearly with bipolar disorder or mixed states can be excluded.

The THINC-it tool consisted of four objective, previously validated cognitive tests (i.e., Symbol Check, Codebreaker, Trails, and Spotter), and one subjective measure of cognition was evaluated through the Perceived Deficits Questionnaire for Depression–5-items (PDQ-5-D). The Spotter was used to assess attention/alertness. The Symbol Check assessed the participants’ working memory and attention. The Codebreaker was modelled on the digitsymbol substitution test (DSST) to measure attention and processing speed. The Trails test was designed to assess elements of executive function. In addition, the PDQ-5-D was used to subjectively measure the participants’ cognitive deficits across five items, which assessed their attention/concentration, planning/organization, and retrospective and prospective memory.

The THINC-it tool was developed and presented results from the statistical outcomes. The raw scores of the PDQ-5-D, Spotter, and Trails were positively correlated with the severity of cognitive impairment, and the raw scores of the SymbolCheck and Codebreaker were negatively correlated with cognitive functioning severity. To ensure that the results were consistent across analyses, Spotter, Trails, and PDQ-5-D scores were converted to standard Z scores and multiplied by -1. For calculation of the total THINC-it composite score, each of the THINC-it tasks was assigned a weight of 0.20. Accordingly, a higher score indicated better cognitive function.

The PSQI was used to assess the participants’ sleep quality and duration. A higher Sleep Quality Index total score reflected poorer subjective sleep quality, with a total score > 7 indicating the presence of sleeping problems.

### Statistical analysis

Statistical analyses were performed using SPSS v22.0. Continuous variables were summarized as the means and standard deviations(SDs) or medians and interquartile ranges(IQRs). The normality of distributions were assessed using the K-S test. The t test and ANOVA were used to compare the results between within-group and groups for normally distributed variables. The Kruskal‒Wallis test was used for nonnormally distributed variables. Continuous variables were further analysed to investigate potential risk factors affecting cognitive functions by linear regression. p < 0.05 was considered statistically significant.

## Results

The demographic and clinical characteristics of the study groups are shown in Table 1. A total of 78 MDD patients were recruited, one of whom was excluded due to having a YMRS total score of 7. Individuals who did not complete the full set of objective and subjective THINC-it tasks and any identifiable outliers were also excluded. Ultimately, 75 patients with depression were included for analysis. The MDD group had a mean age of 33.5 years, and the majority of the sample was female (74.3%). The mean years of education was 13.7 years, and half of the participants were never married (50%). The mean age at onset was 30.2 years (SD = 12.4), and the mean number of depressive episodes was 2.2 (SD = 1.6). The mean HAM-D score was 23.8 (SD = 7.1), and the mean HAM-A score was 14.6 (SD = 6.8). Almost all subjects were recruited from an outpatient psychiatric department and were experiencing variable degrees of depression episodes.

**Table 1:** Demographic and Clinical Characteristics of the Study Cohorts

Characteristics	MDD (n = 75)	HC (n = 100)	t/Z	*P*
Age (years, mean ± SD)	33.5 ± 12.1	31.6 ± 6.0	1.313	0.242
Gender (n, %)			-0.405	0.686
Male	18 (25.7)	23 (23.0)		
Female	52 (74.3)	77 (77.0)		
Marital status (n, %)			-0.682	0.390
Never married	35 (50.0)	40 (40.0)		
Married	33 (47.1)	60 (60.0)		
Divorced	2 (2.9)	0 (0.0)		
Occupation(n, %)			-0.785	0.433
Unemployed	3 (4.3)	2 (2.0)		
Housewife/husband	1 (2.9)	4 (4.0)		
Employment	53 (75.7)	78 (78.0)		
Retired	5 (7.1)	0 (0.0)		
Student	7(10.0)	16 (16.0)		
Years of education (years, mean ± SD)	13.7 ± 2.1	15.9 ± 3.6	-4.939	< 0.001^***^
Age at onset (years, mean ± SD)	30.2 ± 12.4			
Duration of depression (weeks)	17.2 ± 22.0			
Disease duration (months)	44.5 ± 59.9			
Number of depressive episodes	2.2 ± 1.6			
Scales scores				
HAM-D scores (mean ± SD)	23.8 ± 7.1	0.4 ± 1.2	27.351	< 0.001^***^
HAM-A scores (mean ± SD)	14.6 ± 6.8	0.3 ± 1.1	17.275	< 0.001^***^
YMRS scores (mean ± SD)	0.3 ± 1.6	3.1 ± 6.0	3.873	< 0.001^***^
PSQI scores (mean ± SD)	11.5 ± 4.1	3.9 ± 2.3	-9.931	< 0.001^***^

Abbreviations: MDD, major depressive disorder; HC, healthy controls, HAM-D, Hamilton Depression Scale; YMRS, Young Mania Rating Scale; SD, standard deviation; HAM-A, Hamilton Anxiety Scale; PSQI, Pittsburgh Sleep Quality Index. Note: ^***^, *P* < 0.001

Based on the rank-sum analysis, cognitive function scores were derived by averaging the Z scores of all the subtests. Significant differences were observed between the two groups in the THINC-it total scores or the Spotter, Codebreaker, Trails, and PDQ-5-D scores (*P* < 0.001). In contrast, no significant difference was observed in the Symbol Check scores (see Table 2).

**Table 2:** Comparison of z scores using the THINC-it tool between the MDD and HC groups

Variables	MDD (n = 75)	HC (n = 100)	Z	*P*	F^a^	*P* ^a^
Spotter	-0.516 ± 1.210	0.440 ± 0.732	-5.319	< 0.001^***^	30.966	< 0.001^***^
Symbol Check	-0.299 ± 0.940	0.094 ± 0.916	-1.040	0.298	0.028	0.868
Codebreaker	-0.400 ± 1.071	0.305 ± 0.759	-4.451	< 0.001^***^	10.811	0.001
Trails	-0.072 ± 0.646	0.291 ± 0.445	-4.173	< 0.001^***^	9.146	0.003
PDQ-5-D	-0.720 ± 0.919	0.539 ± 0.666	-8.034	< 0.001^***^	83.273	< 0.001^***^
THINC-ittotal scores	-0.348 ± 0.636	0.334 ± 0.401	-6.548	< 0.001^***^	45.822	< 0.001^***^

Abbreviations: MDD, major depressive disorder; HC, healthy controls; PDQ-5-D, Perceived Deficits Questionnaire for Depression–5-items. Note: F^a^, P^a^, F, P value after adjusting for years of education; ***, *P* < 0.001

Linear regression models were estimated using the THINC-it total scores, Spotter, Codebreaker, Trails, Symbol Check, and PDQ-5-D as the dependent variables, and age, age at onset, years of education, number of depressive episodes, disease duration, duration of depression as the independent variable. It showed that the THINC-it total scores, Spotter, Codebreaker, Trails, and Symbol Check scores were associated with age and age at onset (P < 0.01). The Codebreaker total scores were positively correlated with years of education (P < 0.05) (Table 3).

**Table 3:** Linear regression between the THINC-it and clinical characteristics

Variables	Age	Years of education	Number of depressive episodes	Disease duration	Age at onset	Duration of depression
Spotter	-4.606^***^	-0.286	0.161	0.177	-4.506^***^	1.410
Symbol Check	-4.065^***^	1.424	-1.482	-0.198	-3.711^***^	-0.012
Codebreaker	-6.284^***^	2.255^*^	0.092	-0.195	-4.976^***^	-0.104
Trails	-4.543^**^	1.867	-0.749	-0.899	-4.344^***^	0.234
PDQ-5-D	0.392	-0.404	1.084	1.255	-0.502	0.545
THINC-it total scores	-6.611^***^	1.523	-0.057	-0.146	-6.052^***^	0.824

Abbreviations: PDQ-5-D, Perceived Deficits Questionnaire for Depression–5-items. Note: *, *P* < 0.05; **,*P* < 0.01; ***, *P* < 0.001

Linear regression showed that the THINC-it total scores, Symbol Check and Codebreaker were associated with the HAM-D and PSQI total scores (*P* < 0.05). The Trails was correlated with HAM-D total scores (*P* < 0.05). The PDQ-5-D was correlated with the PSQI total scores (*P* < 0.05) (Table 4).

**Table 4:** Linear regression between the THINC-it, HAM-D, HAM-A, and PSQI

Variables	HAM-D total scores	HAM-A total scores	PSQI total scores
Spotter	-0.687	0.106	-0.261
Symbol Check	-2.128^*^	-1.032	-2.057^*^
Codebreaker	-2.122^*^	0.293	-2.325^*^
Trails	-2.330^*^	0.134	-0.328
PDQ-5-D	-0.445	-0.924	-2.689^**^
THINC-it total scores	-2.910^**^	-0.966	-2.908^**^

Abbreviations: PDQ-5-D, Perceived Deficits Questionnaire for Depression–5-items; HAM-D, Hamilton Depression Scale; HAM-A, Hamilton AnxietyScale; PSQI, Pittsburgh Sleep Quality Index. Note: *, *P* < 0.05; **,*P* < 0.01; ***, *P* < 0.001

## Discussion

In this study, the THINC-it tool was used as a quick, easy-to-use, and practical screening tool to assess MDD adults’ daily cognitive functions. Previous studies have shown that the corresponding clinical factors affect patients’ cognitive abilities [21]. Thus, a transversal naturalistic study was conducted to highlight different cognitive profiles of patients with specific clinical symptoms of depression.

### Objective and subjective cognitive impairment exists in MDD

A previous study found that cognitive symptoms were pervasive and that MDD patients had impaired cognitive domains such as reduced executive functioning, attention, memory, learning, psychomotor speed, and verbal processing [4, 22, 23]. In the THINC-it tool assessment, a difference between objectiveand subjective cognitive impairment was found in depression, and subjective cognitive impairment was significantly correlated with social dysfunction and anxiety [24, 25]. Previous studies also found that subjective cognitive impairments improve more quickly when depression symptoms are relieved early [26]. Compared to the healthy controls, the MDD patients had significantly different THINC-it total scores and Spotter, Codebreaker, Trails, and PDQ-5-D scores, but no differences were found in the Symbol Check in our study. The results of the present study were consistent with these previous studies and showed that MDD patients had impairments in most cognitive domains, including attention/vigilance, processing of speed, psychomotor speed, executive function, and subjective cognition. However, we did not find evidence of impaired working memory. The observed differences might partly be explained by the use of Symbol Check scores to assess dual tasks. Most of the study subjects, including healthy controls, were easily distracted by multivariate tasking, resulting in lower scores that were indistinguishable.

### Age and age at onset should be considered predictors of objective cognitive impairment

We found that age and age at onset were associated mainly with impairment in cognitive domains such as attention/alertness, processing speed, working memory, and executive function. Previous literature reported that the aggravation of cognitive impairments was associated with increasing age [27], however, the difference remained in the domain of specific cognitive impairment. Rapp et al. [28] showed that depressed patients in both the early-onset and late-onset groups had cognitive impairment, but the late-onset patients demonstrated a wider range of cognitive impairment, with more severe symptoms. From the biological point of view [29], one probable explanation might be the association with increased incidence of vascular disease and the presence of more severe cognitive impairments in elderly patients. Additionally, the results of this study support the difference between objective and subjective cognitions. We chose the THINC-it as a cognitive screening tool due to its wide applicability and found that age and age at onset should be considered predictors of objective but not subjective cognitions, which was consistent with the findings of Srisurapanontet et al. [30]. Neurobiologically, these findings seem rational because objective cognition is mainly determined by brain functions, which are superior to those of younger individuals in terms of physiological functions. However, a previous study indicated that age and severity of depression might predict differences between objective and subjective cognitions [31]. Thus, these observations should still be confirmed in further studies.

### The severity of depressive symptoms and sleep problems were associated with impaired working memory, information processing speed, and executive functioning

In this study, the presence of anxiety symptoms did not appear to be associated with subjective or objective cognitive impairments, but we found that an increase in depression severity was associated with impaired working memory, processing speed, and executive function but not with subjective cognitive impairment, which was concordant with the findings of Cha et al. [20]. However, some scholars held contrasting views and reported a correlation between the severity of depression and subjective cognitive impairment but not with objective cognitive impairment [32–34] and even suggested that the severity of depression was not affected by the performance of the whole cognitive field [35]. On the one hand, the obvious limitations of these works were the small sample size and limited neuropsychological tests, while on the other hand, patients enrolled were generally young, had full-time employment, believed cognitive impairment was equivalent to IQ damage, and subjectively refused to acknowledge the decline in work and social function caused by illness. Patients’ stigma is influenced by their cultural background. As a result of the introversion of traditional Chinese culture, Chinese people are used to suppressing their emotions and desires, often asking people to self-absorptionor and self-demand, but not asking others or trying to change the external environment. However, Western culture have an extroverted nature and are more open to change the environment. Consequently, whether different cultural backgrounds differentially affect subjective and objective cognitive impairment in depression still needs further research. In addition, there is consensus that sleep disorders impair cognitive functions despite the unclear underlying mechanisms. In the present study, we found that sleep problems were an important risk factor for cognitive impairment but did not distinguish between subjective and objective cognitive functions. Some previous research supported this conclusion [36, 37]. Our study used a cross-sectional design and collected only a few data relevant to cognitive impairment. In a recent study [20], it was reported that both sleep problems and depression severity independently predicted subjective cognitive impairment, therefore indicating that these should be viewed with caution and need further confirmation.

### Education as a protective factor in the objectivecognitive domains of processing speed

Our results indicated that education was a protective factor in the objective cognitive domains of processing speed. The previous conclusion indicated that the degree of education represented some level of intelligence and could partially counteract cognitive impairment [38]. In addition, there is evidence showing that the estimated education and intelligence level are protective factors against potential cognitive dysfunction or may even compensate for cognitive competence when the damage has already occurred [39]. Thus, activities such as increasing reading hours, playing logical games, or learning new skills designed to increase patients’ cognitive reserve might be beneficial. In contrast, there is still a need for further validation regarding the use of the THINC-it in patients with low levels of education.

### Limitations

The current study also had some shortcomings. First, only patients from the outpatient department were recruited, hence, the current findings may not be generalized to all MDD Chinese patients. Second, some factors potentially influencing the results, such as occupation and medication, were not taken into account. Third, as this was a cross-sectional study, the longitudinal relationship between cognition, depressive symptoms, and other functional outcomes need further investigation.

## Conclusion

The present study showed that the cognitive dysfunction of depressive disorder was pervasive, and there was a significant relationship between several cognitive domains and specific clinical variables, such asage, age at onset, depressive symptoms, sleeping problems, and education. Education as a protective factor in the objective cognitive domains of processing speed. Our study provides a basis for the routine clinical application of the THINC-it and targeted interventions for risk factors for depression.

## Data Availability

The raw data supporting the conclusions of this article will be made available by the authors (Na Zhu, zhuna1987524@aliyun.com), without undue reservation.
